# Regulation of Rab5 isoforms by transcriptional and post‐transcriptional mechanisms in yeast

**DOI:** 10.1002/1873-3468.12785

**Published:** 2017-08-24

**Authors:** Oliver Schmidt, Yannick Weyer, Matthias J. Fink, Martin Müller, Sabine Weys, Marietta Bindreither, David Teis

**Affiliations:** ^1^ Division of Cell Biology, Biocenter Medical University of Innsbruck Austria

**Keywords:** mRNA decay, Rab5 GTPases, stress, Vps21, Ypt53

## Abstract

Rab5 GTPases are master regulators of early endosome biogenesis and transport. The genome of *Saccharomyces cerevisiae* encodes three Rab5 proteins: Vps21, the major isoform, Ypt52 and Ypt53. Here, we show that Vps21 is the most abundant Rab5 protein and Ypt53 is the least abundant. In stressed cells, Ypt53 levels increase but never exceed that of Vps21. Its induction requires the transcription factors Crz1 and Gis1. In growing cells, the expression of Ypt53 is suppressed by post‐transcriptional mechanisms mediated by the untranslated regions of the *YPT53* mRNA. Based on genetic experiments, these sequences appear to stimulate deadenylation, Pat1‐activated decapping and Xrn1‐mediated mRNA degradation. Once this regulation is bypassed, Ypt53 protein levels surpass Vps21, and Ypt53 is sufficient to maintain endosomal function and cell growth.

## Abbreviations

**ESCRT**, endosomal sorting complexes required for transport

**MVB**, multivesicular body

**TF**, transcription factor

**utr**, untranslated region

**vps**, vacuolar protein sorting (gene)

Small GTPases of the Rab (ras‐related in brain) family regulate vesicular transport in eukaryotic cells. The yeast genome encodes 11 and the human genome more than 60 Rab‐family proteins 1. Rab GTPases localize to membranes through a carboxy‐terminal prenylation motif and exist in an inactive GDP‐bound and an active GTP‐bound state. The GTP loading (activation) is catalysed by guanine nucleotide exchange factors (GEFs). GTPase‐activating proteins (GAPs) stimulate GTP hydrolysis and terminate Rab signalling.

Rab5 proteins are master regulators of early endosome biogenesis and maturation. GTP‐Rab5 interacts with numerous effectors, which function in vesicle tethering, soluble *N*‐ethyl‐maleimide‐sensitive factor attachment protein receptor (SNARE) priming, endosome motility along microtubules and in the regulation of phosphatidylinositol 3‐phosphate (PI(3)P) kinase activity 1, 2. Together they organize domains on endosomes that promote homotypic endosome fusion and maturation. Termination of Rab5 signalling on endosomes and activation of Rab7 enables mature late endosomes to fuse with lysosomes 3. The human and the mouse genomes encode Rab5a, b and c sequences that are about 85% identical. Only when all three Rab5 homologues reached subcritical protein levels, the endosomal system collapses with a marked reduction in the number of early endosomes, late endosomes and lysosomes 4, 5.

*Saccharomyces cerevisiae* Rab5 orchestrates a functionally similar set of effectors to control endosome biogenesis 6, 7. The three isoforms, Vps21, Ypt52 and Ypt53 are 50–60% identical 8. Deletion of *VPS21* impairs cell growth and causes a strong vacuolar protein sorting (*vps*) phenotype with a block in lysosomal (vacuolar) delivery of endocytic, biosynthetic and autophagic cargo 8, 9, 10, 11. Single or double deletions of the minor Rab5 isoforms, *YPT52* or *YPT53,* do not result in overt membrane trafficking or growth defects. Yet, loss of *YPT52* or *YPT53* in a *VPS21* deletion mutant (*vps21*∆) further decreases cell growth. These genetic interactions suggest at least in part specific functions for Vps21, Ypt52 and Ypt53 and only partial redundancy 8, 12. This is supported by differences in their effector proteins. GTP loading of Ypt52 is negatively regulated by the non‐SCF‐type F‐box protein Roy1 together with Skp1, which can sequester Ypt52‐GDP or nucleotide‐free Ypt52 13. Ypt52 has a high GTPase activity even in the absence of any GAP 12. Ypt53 shows low sensitivity towards the Rab5‐GAP Msb3/Gyp3, but higher sensitivity towards several other yeast GAPs *in vitro* 12, 14. Vps9 and Muk1 function as GEFs for all Rab5 isoforms 15, 16. Ypt53 protein levels are low during vegetative growth. This has been attributed to suboptimal codon usage and low transcriptional activity 8, 12. In response to various stress conditions including respiratory growth, entry into stationary phase, nitrogen starvation and growth in the presence of high calcium concentrations Ypt53 protein levels increase 12, 17. During extended nitrogen starvation for days, Ypt53 contributes to the biosynthetic sorting of the vacuolar enzymes Ape1 and Prc1 17. Specific/unique physiological roles for Ypt52 or Ypt53 have not been identified.

To better characterize how yeast cells coordinate the expression and function of the three different Rab5 isoforms, we directly compared their protein levels and found that Ypt53 was least abundant. The transcriptional induction of Ypt53 expression occurs in response to defects in endolysosomal trafficking and nutrient limitation, and required the transcription factors (TFs) Gis1 and Crz1 in a nonredundant manner. Even under the strongest tested induction conditions, Ypt53 levels remained approximately 5–10‐fold lower than Vps21, mainly because the protein levels of Ypt53 were additionally controlled by *YPT53* mRNA decay. Genetic experiments suggest that *YPT53* mRNA abundance was governed by sequences upstream and downstream of the *YPT53* open reading frame, which triggered the deadenylation/decapping‐dependent 5′–3′ mRNA decay pathway. Bypassing this regulation by exchanging 5′ and 3′ sequences allows Ypt53 to reach protein levels of Vps21 and to function as a *bona fide* Rab5 protein. Our findings imply mRNA degradation as a means to control Rab5 isoform expression in budding yeast.

## Materials and methods

### Yeast cell culture

All yeast strains were derivatives of SEY6210 or BY4742 (where indicated). BY4742‐derived strains were from the MATalpha knockout collection (GE Healthcare, Chicago, IL, USA). The *vps8∆* strain was in the BY4738 background, which is isogenic with BY4742 except for *HIS3* ^+^. For growth under standard conditions cells were incubated at 26 °C in YNB synthetic medium supplemented with amino acids and 2% glucose and grown into midlogarithmic phase (OD: 0.4–0.8). For starvation experiments, cells were kept at midlog phase for 24 h before they were twice washed with and resuspended in YNB with 2% glucose but without amino acids and (NH_4_)_2_SO_4_. For growth on agar plates, yeast cells were diluted to OD_600 nm_ = 0.05 and spotted in serial dilutions on selective YNB plates. Protein synthesis was inhibited by treatment with cycloheximide (Merck, Darmstadt, Germany; 50 μg·mL^−1^).

### Yeast strains, plasmids and cloning

Genetic modifications were done by PCR and/or homologous recombination using standard techniques. Plasmid‐expressed genes including their endogenous promoters were amplified from yeast genomic DNA into centromeric vectors (pRS series). Chimeric constructs or point mutations were generated by overlap extension PCR. All constructs were analysed by DNA sequencing and transformed into yeast cells using standard techniques. Yeast strains and plasmids used in this study are listed in Table S1 and primer in Table S2.

### Live cell fluorescence microscopy

A Zeiss Axio Imager M1 (Zeiss, Göttingen, Germany) equipped with standard fluorescent filters and a SPOT Xplorer CCD camera was used. VisiView software was used for image acquisition. Brightness and contrast were linearly adjusted using adobe photo shop CS5 (Adobe Photoshop, San Jose, CA, USA). Mup1‐GFP degradation was induced by treatment with 100 μg·mL^−1^ l‐methionine. For vacuole staining 18, cells were labelled for 10 min with 10 μg·mL^−1^ FM4‐64 (stock solution 1 mg·mL^−1^ in DMSO), washed twice and subsequently chased for 1 h before microscopy was performed.

### Preparation of yeast whole cell protein extracts, western blot and immunodetection

Proteins were extracted by alkaline extraction 20, separated by SDS/PAGE (Biorad, Hercules, CA, USA) and transferred to poly(vinylidene difluoride) membranes (GE Healthcare) with the semidry method. Western blot signals were developed with enhanced chemoluminescence reagent on CL‐XPosure film (Thermo Fisher, Waltham, MA, USA). Films were digitalized on a Perfection‐3200 scanner (Epson, Suwa, Japan). If not otherwise stated, western blot signals were quantified by densiometry using imagej version 1.47T (open access software) 19, normalized to the respective loading controls, and presented as mean ± standard deviation from three independent experiments. In case of strong differences in Vps21/Ypt53 expression levels, in some cases different exposure times had to be used for quantifications to reduce image saturation. In this case, bands with intermediate intensities were used to harmonize the values. Antibodies used in this study include: anti‐Pgk1 (22C5D8; Thermo Fisher), anti‐HA (12CA5, Abcam or 16B12; Covance, Anopoli Biomedical Systems, Eichgraben, Austria), anti‐Vps4, anti‐Snf7 21 and anti‐Vps21 11 were kindly provided by Scott Emr, Cornell University. anti‐Ape1 22 was kindly provided by Claudine Kraft, University of Vienna.

### RNA isolation and quantitative PCR (RT‐qPCR)

Logarithmically growing cells (40 OD) were harvested by centrifugation and immediately frozen in lq. N_2_. Cell pellets were lysed with 1‐mm glass beads in a FastPrep‐24 homogenizer (MP Biomedicals, Santa Ana, CA, USA) in Qiagen RLT buffer, and RNA was extracted using the Qiagen RNeasy Mini kit (Qiagen, Venlo, the Netherlands). Yield and purity were determined photometrically. cDNA was prepared from 5 μg DNAse I‐treated RNA using the RevertAid First strand cDNA synthesis kit (Thermo Fisher) with oligo‐dT primer according to the standard protocol. For analysis of RNA degradation mutants, a 1 : 1 mixture of random hexamer and oligo‐dT primer was used. Quantitative PCR (qPCR) was performed in a 10‐μL scale with 2 μL of cDNA, 5 μL 2xTAQman qPCR mix (Thermo Fisher) and 0.5 μL TAQman probe on a PikoReal 96 Real‐time PCR system (Thermo Fisher) with 7‐min initial denaturation (95 °C) and 40 cycles of 5 s (95 °C) and 30 s (60 °C). TAQman gene expression assays were from Thermo Fisher (*YPT53*: Sc04158331_s1; *VPS21*: Sc04165675_s1; *PGK1*: Sc04104844_s1). All probes and primer anneal within coding sequences. Each RT‐qPCR analysis was done from three or more independent biological samples in three to four technical replicates. Data were analysed with the pikoreal software (version 2.2; Thermo Scientific) with manual threshold adjustment, and relative mRNA abundance was calculated in Microsoft Excel using the ∆∆C_T_ method 23. Statistical comparisons were calculated using the Student *t* test. *P* values are denoted as follows: *P < 0.05, **P < 0.01, and ***P < 0.001.

## Results

### Rab5 proteins are upregulated in cells with compromised endosomal protein sorting

**Figure 1:**
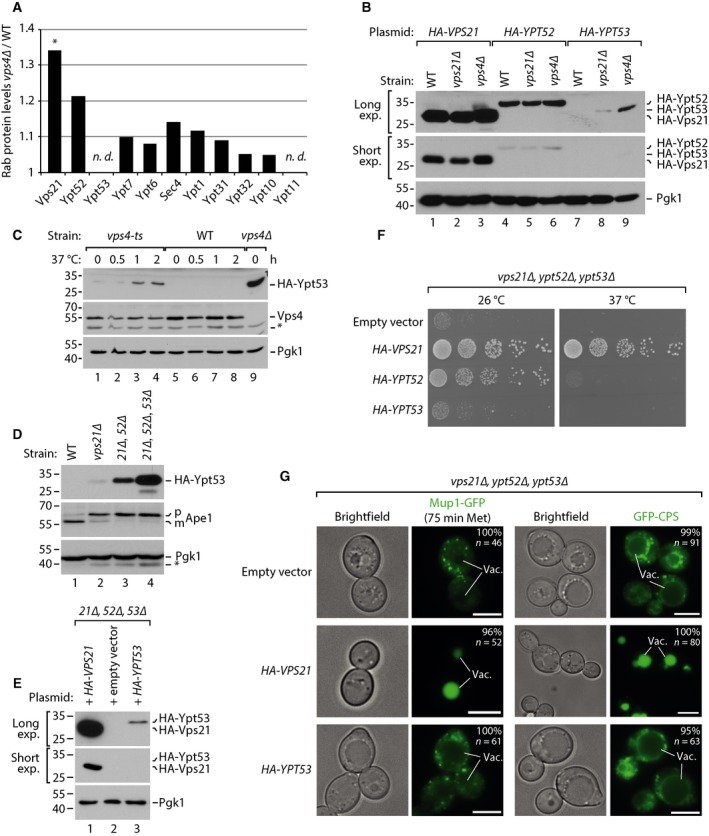
(A) Protein levels of Rab GTPases in WT and *vps4∆* cells (ratio *vps4∆/*WT) deduced from quantitative proteomics 25. *Significant protein ratio changes 25. n.d., not determined. (B–E) SDS/PAGE and western blot of whole cell protein lysates from logarithmically growing cells analysed with the indicated antibodies. (B) WT, *vps4∆* and *vps21∆* cells expressing the indicated Rab5 plasmids. Due to strong differences in expression we provide long and short western blot exposures (exp.). For quantifications see Fig. S2B,C,E,F. (C) WT and *vps4∆* cells expressing *HA‐YPT53* and *vps4‐ts* or empty plasmid grown into logarithmic phase at permissive temperature (26 °C) and shifted to the restrictive temperature (37 °C) for the indicated time. *Unspecific background bands. For quantification see Fig. S2G. (D) WT cells or the indicated mutants expressing *3xHA‐YPT53*. p(recursor) and m(ature) forms of Ape1. For quantification see Fig. S2I. (E) *vps21∆, ypt52∆, ypt53∆* cells expressing the indicated plasmids. (F) Growth of *vps21∆, ypt52, ypt53∆* cells containing centromeric plasmids expressing 3xHA‐tagged *VPS21, YPT52* or *YPT53* from their native promoter and terminator sequences or empty vector at the indicated temperatures. (G) Life cell fluorescence microscopy of *vps21∆, ypt52∆, ypt53∆* cells expressing Mup1‐GFP (after treatment with 100 μg·mL^−1^ l‐methionine for 75 min) or GFP‐CPS and the indicated plasmids.

How yeast cells coordinate the expression of the three Rab5 homologues, Vps21, Ypt52 and Ypt53 is unclear. An extreme situation that requires Vps21 but not Ypt52 is the accumulation of flattened membrane stacks in so called class E compartments 24. These structures typically form – instead of multivesicular bodies (MVBs) – in cells that have defects in the endosomal sorting complexes required for transport (ESCRT). To address how Rab5 protein expression changes, we analysed the quantitative proteome of ESCRT (*vps4∆*) mutants and wild‐type (WT) cells, that we reported earlier 25. Our data set comprised quantitative information for 9 of 11 Rab proteins. Ypt53 and Ypt11 were not quantified, but Vps21 and Ypt52 protein levels were increased moderately (> 1.2‐fold) in *vps4∆* mutants (Fig. 1A).

To directly compare the protein levels of Vps21, Ypt52 and Ypt53, we constructed plasmids expressing N‐terminally 3xHA‐tagged versions of all three genes using their regulatory DNA sequences 5′ (including the promoter) and 3′ (including the terminator) of the open reading frame (orf). All constructs included at least 350 bp upstream and 100 bp downstream of the experimentally determined 5′ and 3′ untranslated regions (utr) of the mRNAs 26. The expression of HA‐Vps21 protein was similar to the endogenous Vps21 (Fig. S1A, S2A) and it was the most abundant Rab5 protein in yeast (Fig. 1B). HA‐Ypt52 protein levels were approximately fivefold lower and HA‐Ypt53 was not or only poorly detected in WT cells (Figs 1B, S2B). Due to these considerable differences in protein expression levels, we were not able to precisely quantify the Vps21:Ypt53 ratio, but estimate Ypt53 levels to be several orders of magnitude lower in WT cells based on western blot analysis. Ypt53 protein levels increased at least 10‐fold in *vps21∆* mutants and also in six different ESCRT mutants (Figs 1B, S1B, S2C,D), although to different extents. We also observed a moderate increase in Vps21 and Ypt52 protein levels in ESCRT mutants, consistent with our proteomic observation (Figs 1A, S1B, S2E,F). The increase of Ypt53 levels in a *vps4‐ts* strain within 30–60 min at 37 °C (Fig. 1C, S2G) was correlated in time with disruption of MVB biogenesis and the development of class E compartments 24. The lytic activity of the vacuole itself does not seem to regulate Ypt53 expression, since loss of its major catabolic activities 25 (Figs S1C, S2H) did not increase cellular Ypt53 protein levels.

In WT cells, GFP‐Vps21 and GFP‐Ypt52 localized to perivacuolar puncta with a diffuse cytoplasmic signal but GFP‐Ypt53 was hardly detected (Fig. S1D). In *vps4∆* mutants, we observed accumulation of GFP‐Vps21, GFP‐Ypt52 and GFP‐Ypt53 on class E compartments (Fig. S1D). Despite the upregulation of Ypt53 in ESCRT mutants and the localization of all Rab5 isoforms to class E compartments, their formation only required Vps21 function (Fig. S1E) as reported before 24.

With the successive deletion of each Rab5 gene (*vps21∆, ypt52∆* and *ypt53∆*) the protein levels of HA‐Ypt53 increased strongly (at least 150‐fold, Figs 1D, S2I) but always remained lower when compared to Vps21 (Fig. 1E). Despite the upregulation, HA‐Ypt53 was not sufficient to rescue growth defects (Fig. 1F), failure of vacuolar protein sorting (Fig. 1G), or pre‐Ape1 maturation caused by defects in selective autophagy via the *cvt* pathway (Fig. 1D) 10. In contrast, expression of HA‐Vps21 was sufficient to complement the growth defect and the temperature sensitivity of a *vps21∆* single mutant or of a *vps21∆, ypt52∆, ypt53∆* triple mutant strain (Fig 1F, S1F) and to restore the transport of the methionine permease Mup1‐GFP into the vacuole upon methionine addition and the biosynthetic transport of GFP‐CPS (Fig 1G). HA‐Ypt52, expressed in addition to endogenous Ypt52, also rescued temperature‐sensitive growth and the defects of *vps21∆* as reported earlier 8, 13 (Fig. S1F,G). It also partially rescued the growth defect of the Rab5 triple mutant at permissive temperature, but not its temperature sensitivity (Fig. 1F).

We then analysed the change in abundance of all three Rab5 isoforms during prolonged nitrogen starvation. The protein levels of HA‐Vps21 remained constant for up to 3 days of starvation (Fig. S1H, S2J). HA‐Ypt52 levels declined during starvation. After 24 h HA‐Ypt53 protein levels were upregulated as reported earlier 17 and increased even further with ongoing starvation, but always remained considerably lower than HA‐Vps21 (compare lanes 4,8,12 in Fig. S1H).

These results suggested that the protein expression levels of different Rab5 isoforms are controlled by nutrient availability and by defects in endosomal trafficking.

### Control of *YPT53* mRNA by transcriptional and post‐transcriptional mechanisms

**Figure 2:**
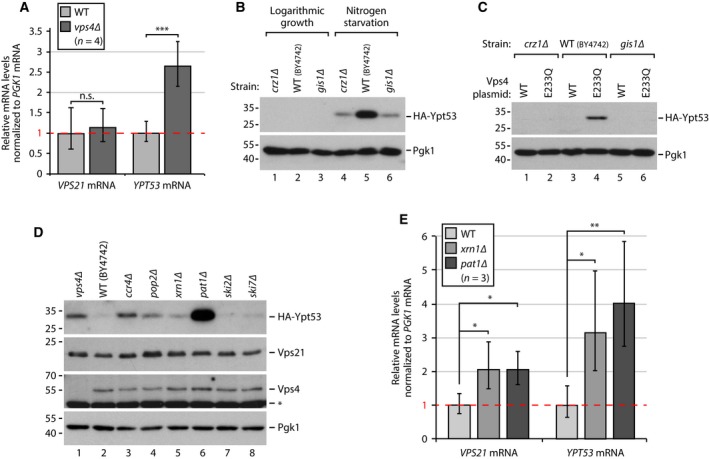
(A) Quantification of *YPT53* and *VPS21* mRNA (normalized to stable *PGK1* mRNA) from logarithmically growing WT (*vps4∆* + pRS413‐*VPS4*) and ESCRT mutant (*vps4∆ *+ pRS413) cells by RT‐qPCR (∆∆C_T_ method), *n* = 4. Error bars indicate standard deviation. n.s., not significant. ***P < 0.001. (B) SDS/PAGE and western blot from whole cell protein lysates of WT (BY4742) and congenic *crz1∆* and *gis1∆* cells in logarithmic phase and after 24 h starvation for amino acids and nitrogen sources analysed with the indicated antibodies. (C) Whole cell protein lysates from logarithmically growing WT (BY4742) cells and the indicated mutants expressing *VPS4* or *vps4* ^E233Q^ from plasmids analysed as in (B). (D) Whole cell protein lysates of logarithmically growing WT (BY4742) and indicated congenic deletion strains analysed as in B). *Nonspecific background bands. For quantifications see Fig. S2K,L. (E) Quantification of *YPT53* and *VPS21* mRNA from logarithmically growing WT (BY4742) and congenic *pat1∆* and *xrn1∆* cells by RT‐qPCR as in A (*n* = 3). *P < 0.05, **P < 0.01.

Next, we analysed *YPT53* and *VPS21* transcripts in WT cells and *vps4∆* mutants by RT‐qPCR. *YPT53* mRNA was 2.6‐fold upregulated in *vps4∆* cells, whereas *VPS21* mRNA was not changed (Fig. 2A). The increase in Ypt53 mRNA could be caused by transcriptional induction or by reduced mRNA decay or by a combination of both mechanisms.

The TFs Crz1 and Gis1 are required for induction of *YPT53* in response to calcium treatment 12 or upon entry into stationary phase 17. Both Gis1 and Crz1 were also indispensable for the efficient upregulation of Ypt53 in response to nitrogen starvation (Fig. 2B) or in cells expressing the dominant‐negative ATP hydrolysis‐deficient allele *vps4* ^*E233Q*^, which disrupts ESCRT function 21 (Fig. 2C).

**Figure 3:**
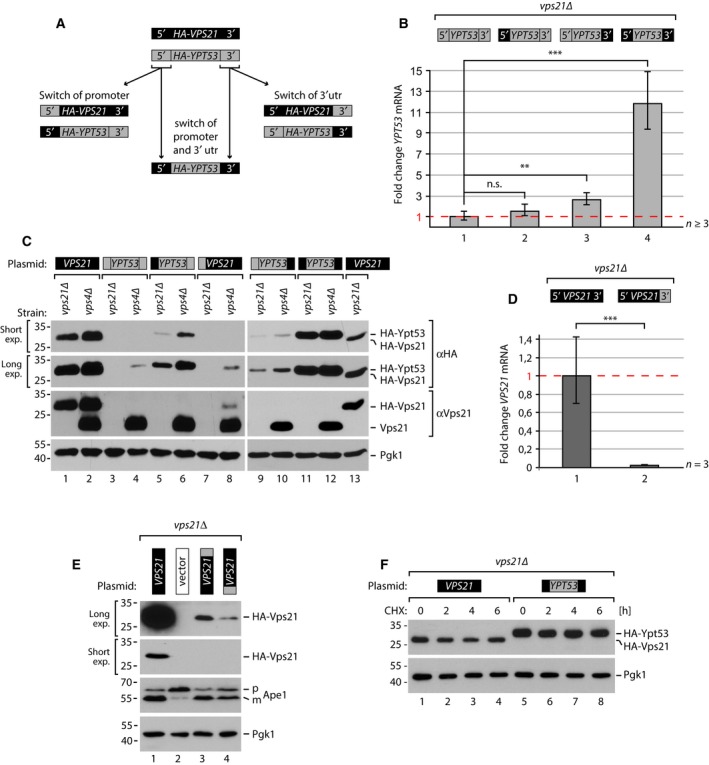
(A) Schematic depicting the chimeric constructs of *VPS21* and *YPT53* with exchanged upstream and downstream genetic regions. (B) Quantification of *YPT53* mRNA normalized to stable *PGK1* mRNA from logarithmically growing *vps21∆* cells expressing the indicated *VPS21/YPT53* chimeric constructs by RT‐qPCR (*n* ≥ 3). Error bars indicate standard deviation. n.s., not significant, **P < 0.01,*** *P* < 0.001 (C) SDS/PAGE and western blot of whole cell protein lysates from logarithmically growing *vps21∆* or *vps4∆* cells expressing the indicated *VPS21/YPT53* chimeric constructs and analysed with the indicated antibodies. Due to strong differences in expression we provide long and short western blot exposures (exp.). For quantification see Fig. S2M. (D) Quantification of *VPS21* mRNA as in (B) (*n* = 3), ***P < 0.001. (E) Whole cell protein lysates from logarithmically growing *vps21∆* cells expressing the indicated *VPS21/YPT53* chimeric constructs and analysed as in (C). p(recursor) and m(ature) forms of Ape1. (F) Whole cell protein lysates of logarithmically growing *vps21∆* cells expressing the indicated plasmids treated with 50 μg·mL^−1^ cycloheximide (CHX) for the indicated times and analysed as in (C). For quantification see Fig. S2N.

Next, we tested how canonical cytoplasmic mRNA decay pathways contribute to the regulation of *YPT53* mRNA abundance. Deadenylation of poly‐A tails by the Ccr4‐Pop2‐Not1 complex is the first step in different mRNA decay pathways. Deadenylated mRNA can be subjected to 3′–5′ degradation by the exosome together with the SKI complex 27. The deletion of two SKI complex subunits (*ski2∆*;* ski7∆)* did not affect HA‐Ypt53 protein levels (Fig. 3D; lanes 7 + 8; Fig. S2K). Alternatively, deadenylated mRNA can recruit the decapping complex to remove the 7‐methylguanosine cap at the 5′ end, which is often facilitated by the adaptor protein Pat1 28. The Pat1‐dependent decapping process enables 5′–3′ mRNA degradation by the exonuclease Xrn1 and additionally inhibits translation initiation 29. *VPS21* and *YPT53* transcripts were both increased in *pat1∆* and *xrn1∆* mutants, but a more pronounced effect on *YPT53* mRNA (three‐ to fourfold) than on *VPS21* mRNA (twofold) was observed (Fig. 2E). Consistently, Ypt53 protein levels were increased in *ccr4∆, pop2∆, xrn1∆* and most strongly in *pat1∆* mutants (Fig. 2D, S2K,L), while Vps21, Vps4 or Pgk1 protein levels were less or not affected.

Based on genetic evidence it seems that *YPT53* transcription is controlled by Crz1 and Gis1. Deadenylation, Pat1‐dependent decapping and 5′–3′ decay of the *YPT53* mRNA additionally repress Ypt53 protein levels.

### *YPT53* expression is negatively regulated by 5′ and 3′ sequence elements

To test how transcriptional regulation and mRNA decay control Ypt53 protein levels, we exchanged the 5′ and 3′ flanking sequences of *HA‐VPS21* and *HA‐YPT53* constructs including their experimentally determined utrs 26 (Fig. 3A). *YPT53* mRNA expressed from the *VPS21* promoter only marginally increased as long as the *YPT53* 3′ utr was still present (Fig. 3B, bar 2), and HA‐Ypt53 protein levels remained considerably lower than HA‐Vps21 (Fig. 3C, lanes 1 + 5; Fig S2M). mRNA and protein levels also increased when the *YPT53* 3′ utr was replaced with 3′ utr of *VPS21* (Fig. 3B, bar 3; Fig. 3C, lanes 1 + 9). Conversely, the transfer of the *YPT53* 3′ utr onto *VPS21* caused a dramatic decrease in *VPS21* mRNA (Fig. 3D) and HA‐Vps21 protein levels (Fig. 3E, compare lanes 1 + 3). Replacing the promoter and 3′ utr of *YPT53* simultaneously with the promoter and the 3′ utr of *VPS21* resulted in a more than 10‐fold increase in *YPT53* mRNA (Fig. 3B, bar 4). Now the protein levels of Ypt53 were higher when compared to Vps21 (Fig. 3C, lanes 11 + 13, Fig. S2M). The protein stability of Vps21 and Ypt53 was also similar. Vps21 has been reported to be a stable protein with a half‐life of > 5 h 30. Upon treatment with cycloheximide for 6 hrs Vps21 but also Ypt53 protein levels barely decreased (Fig. 3F, Fig. S2N).

Taken together, these results indicate that the protein levels of Ypt53 are mainly controlled by 5′ and 3′ sequences mediating mRNA transcription and decay, but not by protein stability.

### Ypt53 can function as the only Rab5 in yeast

**Figure 4:**
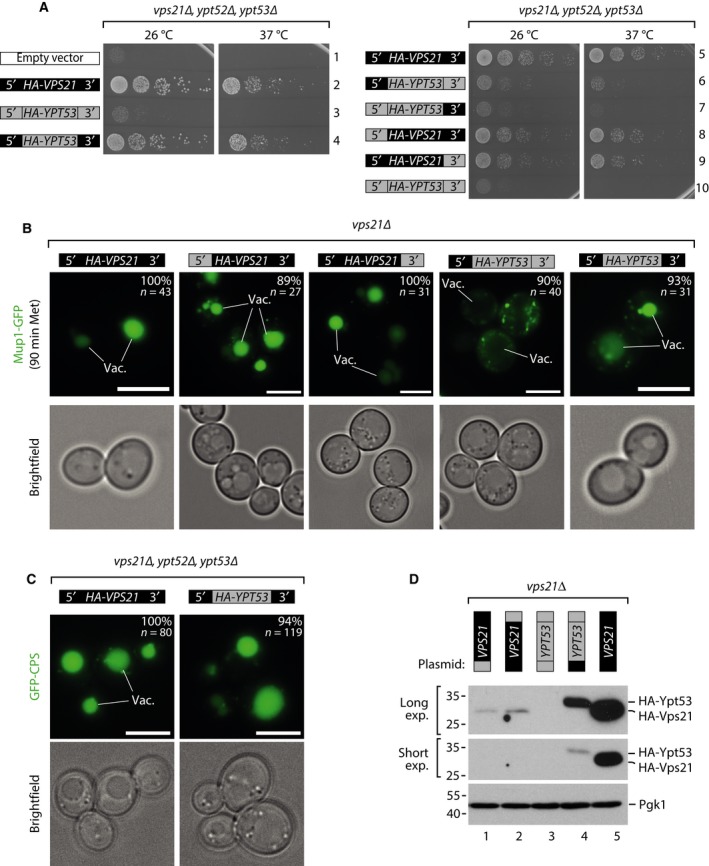
(A) Growth of *vps21∆, ypt52, ypt53∆* cells expressing the indicated constructs at the indicated temperatures. (B) Life cell fluorescence microscopy of Mup1‐GFP in *vps21∆* cells expressing the indicated constructs grown into logarithmic phase and exposed to L‐methionine (100 μg·mL^−1^) for 90 min. Vac(uoles). Size bars 5 μm. (C) Life cell fluorescence microscopy of *vps21∆, ypt52∆, ypt53∆* cells expressing GFP‐CPS and the indicated plasmids. Vac(uoles). Size bars 5 μm. (D) SDS/PAGE and western blot of whole cell protein lysates of logarithmically growing *vps21∆* cells expressing the indicated *VPS21/YPT53* chimeric constructs and analysed with the indicated antibodies.

We tested how changes in the expression levels of Rab5 proteins contributed to their role as master regulators of endosomal biogenesis. When HA‐Ypt53 was expressed using the 5′ and 3′ regulatory sequences of *VPS21,* Ypt53 protein expression levels exceeded Vps21 (Fig. 3C). Under these conditions Ypt53 was sufficient to fully complement growth and sorting defects of *vps21*∆ single or *vps21*∆*, ypt52*∆*, ypt53*∆ triple mutants (Fig. 4A–C). Lower levels of Ypt53 failed to rescue the growth of the triple mutant and vacuolar sorting defects (Fig 4A,B,D). Conversely, Vps21 expressed at very low protein levels (Fig. 3C,E; *HA‐VPS21* constructs with 5′ or 3′ regulatory sequences of *YPT53)* was sufficient to rescue the growth of the Rab5 triple deletion mutant (Fig. 4A, rows 8 + 9), as well as vacuolar sorting of Mup1‐GFP (Fig. 4B).

When strongly overexpressed using the *TDH3* promoter and a heterologous 3′ sequence (Fig. S3A–C), Vps21 and also Ypt53 similarly induced perivacuolar clustering of endosomes 31, 32, which was dependent on the Rab5 effectors Vps8 and Vps9 (Fig. S3D). Thus, overexpressed Ypt53 probably can employ a similar set of accessory molecules as Vps21.

In summary, Ypt53 is able to exert all functions of the major Rab5 isoform Vps21 as reported earlier 12, 17, provided it is expressed at protein levels close to or above native Vps21 expression levels. However, at very low expression levels, only Vps21 but not Ypt53 can maintain sufficient Rab5 activity to promote growth and endosomal protein sorting.

## Discussion

Our findings add a new layer of regulation for Ypt53, a Rab5 isoform in yeast. Ypt53 is present at very low levels because its expression is repressed by mRNA decay. Ypt53 is induced by nutrient starvation and endosomal dysfunction. Ypt52 has higher protein levels but is rendered inactive by sequestration 13. The physiological role of the negative regulation of the ‘minor’ Rab5 isoforms remains an open question.

In early diverged protozoans (kinetoplastida) two Rab5 isoforms are differentially expressed and control different modes of endocytosis 33, 34, 35. In mammals, Rab5 isoforms share a high degree of sequence identity and are probably largely redundant 4, 5. Total Rab5 protein levels could be reduced by 80% before effects on the endosomal system became apparent, suggesting that Rab5 function is resilient to large fluctuations 5. Only few isoform‐specific functions have been described. Rab5c is dispensable for epidermal growth factor receptor trafficking 36 and was implicated in Rac‐mediated motility 37.

Yeast also has 3 Rab5 proteins, but the isoforms Vps21, Ypt52 and Ypt53 are more diverged. Genetic experiments suggest only partial redundancy 8, 12, 17, but specific functions have not been identified. Active Vps21 – the major Rab5 protein in yeast – is able to form heterotypic membrane tethers *in vitro* with active Ypt53, but not with Ypt52 32. It is not clear whether this is due to the high intrinsic inactivation of Ypt52 or to unique structural features of this isoform. Both minor Rab5 isoforms differ from Vps21 in their dependence on GEF activity and in their sensitivity towards Rab5 GAPs. In particular, Ypt53 is less efficiently inactivated by the canonical Rab5 GAP Msb3/Gyp3, but can instead be engaged by a number of other GAPs *in vitro* 12, 14. Thus, dynamics of activation and inactivation *in vivo* could be different from those of Vps21. Under normal growth this might be undesirable and therefore minor Rab5 isoforms are suppressed. In case of Ypt52 this is achieved predominantly post‐translational via the noncanonical F‐box protein Roy1, which sequesters inactive Ypt52 in a membrane‐bound complex 13. In contrast, Ypt53 is suppressed already at the transcript level.

We demonstrate that Vps21 is the most abundant and Ypt53 the least abundant Rab5 protein in yeast. This had been predicted earlier based on theoretical codon usage and indirect assays 8, 17. We show that the induction of Ypt53 requires the TFs Crz1 and Gis1 in a nonredundant manner. Crz1 is the major calcium‐responsive TF in yeast 38. Calcium‐induced transcription of *YPT53* via Crz1 was previously demonstrated 12, 39. Gis1 mediates gene expression upon diauxic shift and entry into G_0_ (stationary phase) 40, and promotes *YPT53* expression in postlog cells 17. Also the induction of Ypt53 in endosomal sorting mutants requires Crz1 and Gis1. How expression of Ypt53 is triggered in *vps* mutants remains unclear but might indicate significant stress signalling in response to defects in endosomal trafficking.

The promoter region of *YPT53* contains a putative calcineurin‐dependent response element (CDRE; consensus sequence: AGCCNC) 41 and a stress‐response element (STRE; AGGGG) 148 and 168 base pairs upstream of the orf. STREs are typically operated by the general stress‐responsive Msn2/4 TFs, but can also be engaged by Gis1 42. Thus, Msn2/4 might also regulate stress‐induced *YPT53* expression, although Msn2 was not detected at the *YPT53* locus upon oxidative stress 43 and Crz1 was found to act as a negative regulator of Msn2‐dependent transcription on STREs 44. Therefore, it seems more likely that Gis1 and Crz1 cooperatively act on *YPT53* expression. Cooperativity of TFs on promoters is a common phenomenon 45, 46, but has never been reported for Crz1 and Gis1.

The regulation of the endolysosomal systems takes a central role in eukaryotes to preserve cellular homoeostasis under nutrient stress conditions. In mammalian cells this is achieved by TFEB, a TF inducing lysosomal and autophagy genes 47, 48. In yeast, several endocytic and vacuolar proteins are upregulated during starvation 25, 49 to boost the endolysosomal system in response to metabolic changes. Which TFs drive endolysosomal adaptations in yeast, is currently unknown. Based on the results of our genetic experiments we speculate that Gis1 and Crz1 may be involved.

Human Rab5 genes are also subject to transcriptional regulation 50, 51, and their mRNAs are repressed by miRNA interference 52, 53, 54. Despite the strong upregulation of Ypt53 in response to stress, its protein levels never reached the proteins levels of Vps21. We find that at least in part this was mediated post‐transcriptionally through the 5′ and 3′ utr of the *YPT53* mRNA. Repression involved canonical mRNA decay pathways, including deadenylation by the Ccr4‐Pop2‐Not1 complex, a Pat1‐dependent step (presumably 5′ decapping) and subsequent 5′ > 3′ degradation by Xrn1. Pat1 is an RNA‐binding protein that often binds to the 3′utr of its targets. The paramount effect of *PAT1* deletion on Ypt53 levels (mRNA and protein) suggests that impairment of translation initiation through 5′‐decapping of the mRNA could also contribute to *YPT53* repression.

Artificially bypassing these regulatory mechanisms and increasing Ypt53 protein levels above those of Vps21 allows Ypt53 to function as the sole Rab5 protein in yeast. This is consistent with earlier work that found that overexpression of Ypt53 was sufficient to complement the endosomal defects of a *vps21∆, ypt52∆*, double mutant 12 or a Rab5 triple mutant 17. Whether conditions exist under physiological settings that allow Ypt53 to function as the major Rab5 activity (e.g. Ypt53 levels approaching Vps21 and/or Vps21 being inactive) remains an open question. Vps21 is capable of maintaining endosomal function even if its protein levels are dramatically reduced. However, under certain stress conditions or when the normal regulation of endosomal sorting is compromised, activation of minor isoforms might allow for subtle alterations in Rab5 function, which could help to sustain endosomal transport. We speculate that under certain conditions bypassing the negative regulatory mechanisms, which normally suppress the minor Rab5 isoforms, helps to buffer fluctuations in the endosomal system and maintain endosomal function in response to different stressors.

## Author contributions

OS and DT conceived and supervised the study and analysed data and wrote the manuscript with contributions from all authors; OS, MJF, YW and SW performed experiments; MM and MB provided and validated tools.

## Supporting information


**Fig. S1.** Related to Fig. 1: (A) SDS/PAGE and western blot from WT and *vps21∆* cells expressing *HA‐VPS21* from plasmid analysed with the indicated antibodies. For quantification see Fig. S2A. (B) Whole cell protein lysates of logarithmically growing WT (BY4742) and congenic ESCRT mutant cells expressing *HA‐YPT53* analysed as in (A). For quantification see Fig. S2D. (C) Whole cell protein lysates of logarithmically growing WT and congenic *vps4∆* and *pep4∆, prb1∆, prc1∆* cells analysed as in (A). For quantification see Fig. S2H. (D) Life cell fluorescence microscopy of FM4‐64‐labelled WT and *vps4∆* cells expressing centromeric plasmids encoding GFP‐tagged Rab5 isoforms (Vps21, Ypt52, Ypt53) from their native promoters/terminators. Exposure times GFP‐Vps21: 500 ms; GFP‐Ypt52: 1000 ms; GFP‐Ypt53: 2000 ms. Vac(uoles); class E compartments. Size bars 5 μm. (E) Life cell fluorescence microscopy of GFP‐CPS in the indicated strains at logarithmic growth. Vac(uoles); class E compartments. Size bars 5 μm. (F) Growth of *vps21∆* expressing the indicated plasmids at the indicated temperatures. (G) Life cell fluorescence microscopy of Mup1‐GFP in *vps21∆* cells expressing the indicated plasmids grown into logarithmic phase and exposed to 100 μg·mL^−1^ l‐methionine for 90 min. Vac(uoles). Size bars 5 μm. (H) SDS/PAGE and western blot of whole cell protein lysates from the indicated strains grown into logarithmic phase (*t* = 0) and then starved for amino acids and nitrogen sources for the indicated time. For quantification see Fig. S2J.

**Fig. S2.** Related to Fig. 1, 2, 3 and S1: (A)‐(N) Quantification of western blot experiments. HA or Vps21 signals (as indicated) were normalized to loading control (Pgk1) and presented as mean ± standard deviation relative to the respective control sample. (A) Quantification of Fig. S1A (*n* = 4, two biological and two technical replicates). (B), (C) Quantification of Fig. 1B (*n* ≥ 3 biological replicates). n.d., not determined. (D) Quantification of Fig. S1B (*n* = 4, two biological and two technical replicates). (E), (F) Quantification of Fig. 1B (*n* = 4, two biological and two technical replicates). (G) Quantification of Fig. 1C (*n* = 4, two biological and two technical replicates). (H) Quantification of Fig. S1C (*n* = 4, two biological and two technical replicates). (I) Quantification of Fig. 1D (*n* = 3 biological replicates). (J) Quantification of Fig. S1H. HA signal normalized to Pgk1 loading control (day 0: *n* = 3 biological replicates; day 3: *n* = 4, two biological and two technical replicates). (K) Quantification of HA‐Ypt53 in Fig. 2D (*n* ≥ 3 biological replicates). (L) Quantification of Vps21 in Fig. 2D (*n* = 4, two biological and two technical replicates). **(M)** Quantification of Fig. 3C (*vps21∆* samples: *n* ≥ 3 biological replicates; *vps4∆* samples: *n* = 4, two biological and two technical replicates). (N) Quantification of Fig. 3F (*n* = 3 biological replicates).

**Fig. S3.** related to Fig. 4. (A) Schematic showing the *VPS21* and *YPT53* overexpression constructs containing identical 5′ (*TDH3* promoter) and 3′ sequences. **(B)** growth of *vps21∆, ypt52, ypt53∆* cells overexpressing *VPS21* or *YPT53* on selective minimal medium at the indicated temperatures. (C) Whole cell protein lysates of logarithmically growing WT, *vps21∆* or *vps4∆* cells overexpressing *VPS21* or *YPT53* analysed as in A). (D) Life cell fluorescence microscopy of Mup1‐GFP in WT, *vps8∆* or *vps9∆* cells overexpressing *VPS21* or *YPT53* before and after treatment with 100 μg·mL^−1^ l‐methionine for 60 min. arrowheads indicated endosome clusters; Vac(uoles); size bars 5 μm.

**Table S1.** List of yeast strains and plasmids.

**Table S2.** Primer sequences.
