# Supplementary figures and images for: Regulation of Rab5 isoforms by transcriptional and post‐transcriptional mechanisms in yeast
Source: FEBS Lett. 2017 Aug 24;591(18):2803–15. doi: 10.1002/1873-3468.12785 (PMC5637908; doi:10.1002/1873-3468.12785)

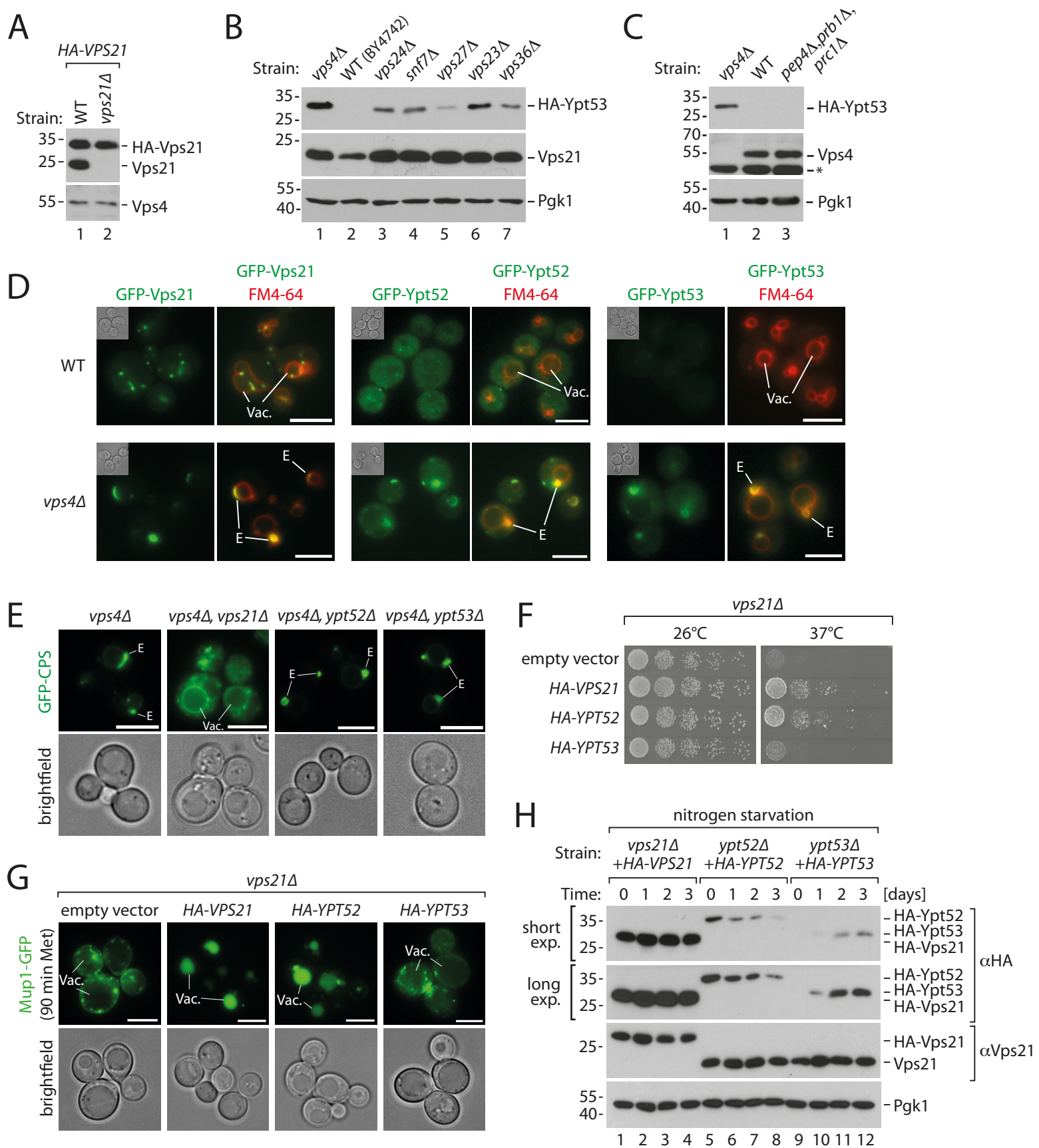

Figure S1

Supplement: Supplementary file 1 — Fig. S1. Related to Fig. 1: (A) SDS/PAGE and western blot from WT and vps21∆ cells expressing HA‐VPS21 from plasmid analysed with the indicated antibodies. For quantification see Fig. S2A. (B) Whole cell protein lysates of logarithmically growing WT (BY4742) and congenic ESCRT mutant cells expressing HA‐YPT53 analysed as in (A). For quantification see Fig. S2D. (C) Whole cell protein lysates of logarithmically growing WT and congenic vps4∆ and pep4∆, prb1∆, prc1∆ cells analysed as in (A). For quantification see Fig. S2H. (D) Life cell fluorescence microscopy of FM4‐64‐labelled WT and vps4∆ cells expressing centromeric plasmids encoding GFP‐tagged Rab5 isoforms (Vps21, Ypt52, Ypt53) from their native promoters/terminators. Exposure times GFP‐Vps21: 500 ms; GFP‐Ypt52: 1000 ms; GFP‐Ypt53: 2000 ms. Vac(uoles); class E compartments. Size bars 5 μm. (E) Life cell fluorescence microscopy of GFP‐CPS in the indicated strains at logarithmic growth. Vac(uoles); class E compartments. Size bars 5 μm. (F) Growth of vps21∆ expressing the indicated plasmids at the indicated temperatures. (G) Life cell fluorescence microscopy of Mup1‐GFP in vps21∆ cells expressing the indicated plasmids grown into logarithmic phase and exposed to 100 μg·mL−1 l‐methionine for 90 min. Vac(uoles). Size bars 5 μm. (H) SDS/PAGE and western blot of whole cell protein lysates from the indicated strains grown into logarithmic phase (t = 0) and then starved for amino acids and nitrogen sources for the indicated time. For quantification see Fig. S2J. [file FEB2-591-2803-s001.pdf]

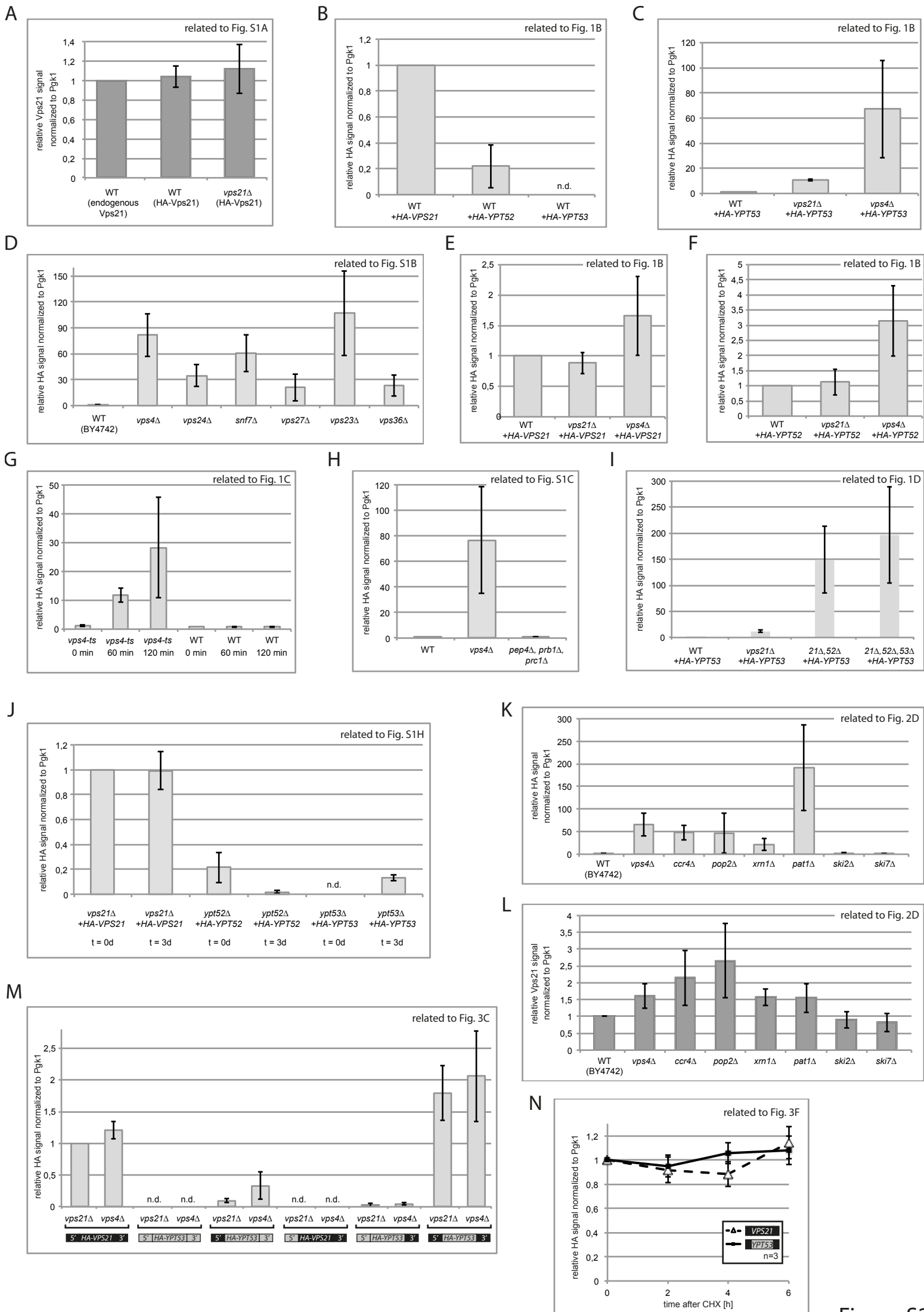

Figure S2

Supplement: Supplementary file 2 — Fig. S2. Related to Fig. 1, 2, 3 and S1: (A)‐(N) Quantification of western blot experiments. HA or Vps21 signals (as indicated) were normalized to loading control (Pgk1) and presented as mean ± standard deviation relative to the respective control sample. (A) Quantification of Fig. S1A (n = 4, two biological and two technical replicates). (B), (C) Quantification of Fig. 1B (n ≥ 3 biological replicates). n.d., not determined. (D) Quantification of Fig. S1B (n = 4, two biological and two technical replicates). (E), (F) Quantification of Fig. 1B (n = 4, two biological and two technical replicates). (G) Quantification of Fig. 1C (n = 4, two biological and two technical replicates). (H) Quantification of Fig. S1C (n = 4, two biological and two technical replicates). (I) Quantification of Fig. 1D (n = 3 biological replicates). (J) Quantification of Fig. S1H. HA signal normalized to Pgk1 loading control (day 0: n = 3 biological replicates; day 3: n = 4, two biological and two technical replicates). (K) Quantification of HA‐Ypt53 in Fig. 2D (n ≥ 3 biological replicates). (L) Quantification of Vps21 in Fig. 2D (n = 4, two biological and two technical replicates). (M) Quantification of Fig. 3C (vps21∆ samples: n ≥ 3 biological replicates; vps4∆ samples: n = 4, two biological and two technical replicates). (N) Quantification of Fig. 3F (n = 3 biological replicates). [file FEB2-591-2803-s002.pdf]

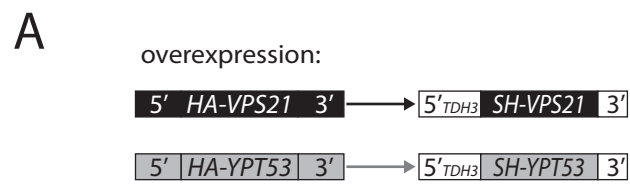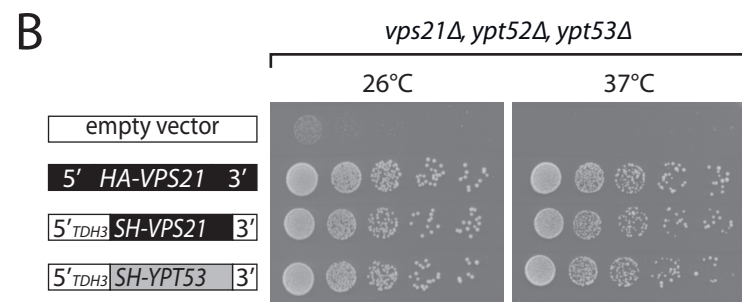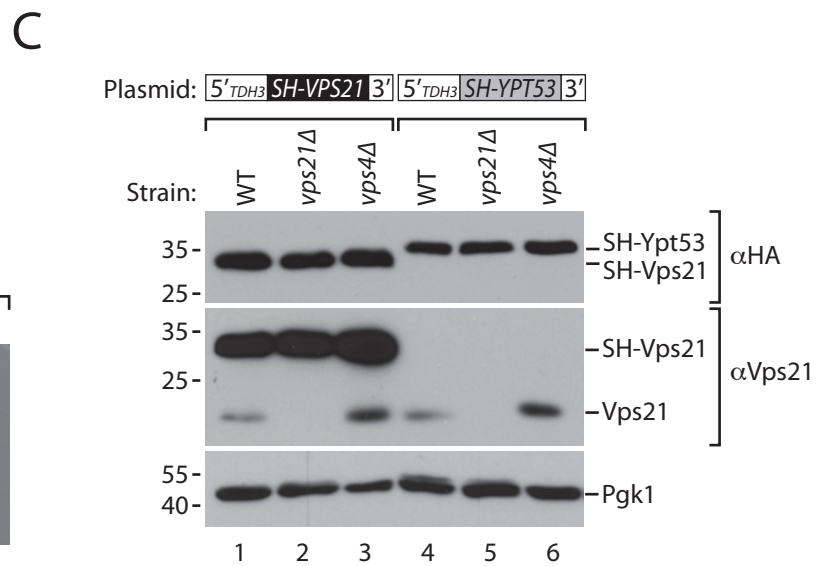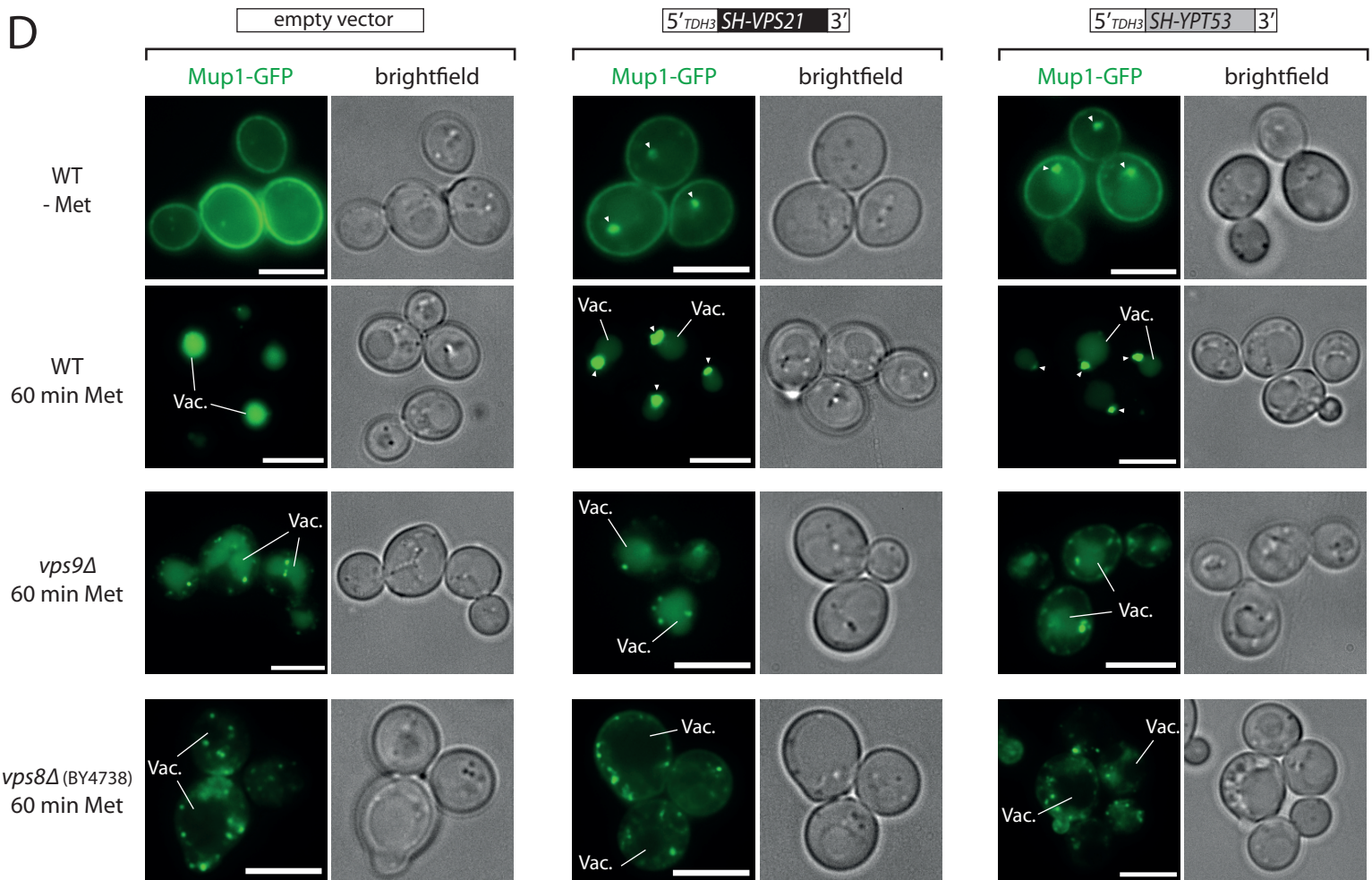

Figure S3

Supplement: Supplementary file 3 — Fig. S3. related to Fig. 4. (A) Schematic showing the VPS21 and YPT53 overexpression constructs containing identical 5′ (TDH3 promoter) and 3′ sequences. (B) growth of vps21∆, ypt52, ypt53∆ cells overexpressing VPS21 or YPT53 on selective minimal medium at the indicated temperatures. (C) Whole cell protein lysates of logarithmically growing WT, vps21∆ or vps4∆ cells overexpressing VPS21 or YPT53 analysed as in A). (D) Life cell fluorescence microscopy of Mup1‐GFP in WT, vps8∆ or vps9∆ cells overexpressing VPS21 or YPT53 before and after treatment with 100 μg·mL−1 l‐methionine for 60 min. arrowheads indicated endosome clusters; Vac(uoles); size bars 5 μm. [file FEB2-591-2803-s003.pdf]
