# Supplementary material for: Regulation of Rab5 isoforms by transcriptional and post‐transcriptional mechanisms in yeast
Source: FEBS Lett. 2017 Aug 24;591(18):2803–15. doi: 10.1002/1873-3468.12785 (PMC5637908; doi:10.1002/1873-3468.12785)
Supplement: Supplementary file 4 — Table S1. List of yeast strains and plasmids. [file FEB2-591-2803-s004.pdf]

Table 1: List of yeast strains and plasmids

strain name	genotype	reference
SEY6210	*MATalpha leu2-3, 112 ura3-52 his3-200 trp1-901 lys2-801 suc2-9*	Robinson et al., 1988
SEY6210.1	*MATa leu2-3, 112 ura3-52 his3-200 trp1-901 lys2-801 suc2-9*	Robinson et al., 1988
MBY3	SEY6210 *vps4::TRP1*	Babst et al., 1997
MBY4	SEY6210.1 *vps4::TRP1*	Babst et al., 1997
MPY93	SEY6210 *vps21::HIS3*	Horazdovsky et al., 1997
DTY515	SEY6210.1 *vps21::HIS3*	this study
MPY96	SEY6210.1 *vps4::TRP1, vps21::HIS3*	this study
MPY99	SEY6210.1 *ypt52::HIS3*	this study
MPY100	SEY6210.1 *vps4::TRP1, ypt52::HIS3*	this study
DTY508	SEY6210.1 *ypt53::HIS3*	this study
DTY509	SEY6210.1 *vps4::TRP1, ypt53::HIS3*	this study
DTY514	SEY6210.1 *vps21::HIS3, ypt52::HIS3, ypt53::HIS3*	this study
DTY518	SEY6210.1 *vps21::HIS3, ypt52::HIS3*	this study
DTY516	SEY6210 *vps21::HIS3, ypt52::HIS3, ypt53::HIS3*	this study
MPY101	SEY6210 *vps21::HIS3, ypt52::HIS3*	this study
MPY99	SEY6210.1 *ypt52::HIS3*	this study
DTY508	SEY6210.1 *ypt53::HIS3*	this study
MMY10	SEY6210.1 *MUP1-GFP::HIS3*	this study
SSY1	SEY6210.1 *vps9::HIS3*	this study
TVY614	SEY6210 *pep4::LEU2; prc1::HIS3; prb1::HisG*	Gerhardt et al., 1998
BY4742	*MATalpha his3Δ 1; leu2Δ 0; lys2Δ 0; ura3Δ 0*	Brachmann et al., 1998
OSY023	BY4742; *vps4::KanMX4*	Open Biosystems
OSY021	BY4742; *vps24::KanMX4*	Open Biosystems
OSY022	BY4742; *snf7::KanMX4*	Open Biosystems
OSY018	BY4742; *vps27::KanMX4*	Open Biosystems
OSY019	BY4742; *vps23::KanMX4*	Open Biosystems
OSY020	BY4742; *vps36::KanMX4*	Open Biosystems
OSY182	BY4742; *crz1::KanMX4*	Euroscarf
OSY430	BY4742; *gis1::KanMX4*	Open Biosystems
OSY480	BY4738 (BY4742 HIS3^{+}); *vps8::KanMX4*	Open Biosystems
OSY389	BY4742; *ccr4::KanMX4*	Open Biosystems
OSY390	BY4742; *pop2::KanMX4*	Open Biosystems
OSY388	BY4742; *xrn1::KanMX4*	Open Biosystems
MFY01	BY4742; *pat1::KanMX4*	Open Biosystems
OSY384	BY4742; *ski2::KanMX4*	Open Biosystems
OSY385	BY4742; *ski7::KanMX4*	Open Biosystems

plasmid name	genotype	reference
pRS413	empty centromeric vector HIS3	Sikorski and Hieter, 1989
pRS414	empty centromeric vector TRP1	Sikorski and Hieter, 1989
pRS415	empty centromeric vector LEU2	Sikorski and Hieter, 1989
pRS416	empty centromeric vector URA3	Sikorski and Hieter, 1989
pOS139	pRS416 - *GFP-VPS21* (endogenous 5' and 3')	this study
pMP29	pRS416 - *GFP-YPT52* (endogenous 5' and 3')	this study
pOS079	*pRS416 - GFP-YPT53* (endogenous 5' and 3')	this study
pOS031	pRS415 - ^{TDH3}*GFP-CPS*	this study
pMP8	pRS416 - *3xHA-VPS21* (endogenous 5' and 3')	this study
pMP9	pRS416 - *3xHA-YPT52* (endogenous 5' and 3')	this study
pMP16	pRS416 - *3xHA-YPT53* (endogenous 5' and 3')	Müller et al., 2015
pMA42	pRS415 - *vps4-ts*	Adell et al., 2014
pMB4	pRS413 - *VPS4*	Babst et al., 1997
pOS014	pRS415 - *VPS4*	Müller et al., 2015
pOS015	pRS415 - *vps4*^{E233Q}	Adell et al., 2014
pOS065	pRS416 - $^{5'\ VPS21}$*3xHA-YPT53*$^{3'\ YPT53}$	this study
pOS069	pRS416 - $^{5'YPT53}$*3xHA-YPT53*$^{3'\ VPS21}$	this study
pOS066	pRS416 - $^{5'YPT53}$*3xHA-VPS21*$^{3'\ VPS21}$	this study
pMF01	pRS416 - $^{5'\ VPS21}$*3xHA-VPS21*$^{3'\ YPT53}$	this study
pOS068	pRS416 - $^{5'\ VPS21}$*3xHA-YPT53*$^{3'\ VPS21}$	this study
pCHL642	pRS416 - *MUP1-GFP*	Lin et al., 2008
pMM02	pRS415 - *MUP1-GFP*	this study
pMP30	pRS416 - ^{TDH3}*SH-VPS21*	this study
pMP31	pRS416 - ^{TDH3}*SH-YPT53*	this study
