# Supplementary material for: Regulation of Rab5 isoforms by transcriptional and post‐transcriptional mechanisms in yeast
Source: FEBS Lett. 2017 Aug 24;591(18):2803–15. doi: 10.1002/1873-3468.12785 (PMC5637908; doi:10.1002/1873-3468.12785)
Supplement: Supplementary file 5 — Table S2. Primer sequences. [file FEB2-591-2803-s005.pdf]

Table S2: Primer sequences

primer name:	sequence:
***VPS21* constructs**	
Xho1 Prom Ypt51 fw	gagactcgagGAGTAGAAGATACAGAGATGACTTG
Hind3UTR Ypt51 rev	tctcaagcttCAATATTAACTAATCCATCTCCCGA
YPT51-HA rev	TCAGGAACATCGTATGGGTACATTTTTTTGTGTGATAAAAGGG
YPT51-HA fw	CCCTTTTATCACACAAAAAAATGTACCCATACGATGTTCCTGA
HA-51rev	GAAGTGACTGATGTGTTCATAGCGTAATCTGGAACGTCAT
HA-51 fw	ATGACGTTCCAGATTACGCTATGAACACATCAGTCACTTC
VPS21-orf_EcoRI-F	cgcggaattcATGAACACATCAGTCAC
VPS21-orf_HindIII-R	gcgcaagcttCTAACAACTGCAAGCACTG
Prom21-GFP-F	CCCTTTTATCACACAAAAAAATGAAAGGAGAAGAACTTTTCACTGG
Prom21-GFP-R	CCAGTGAAAAGTTCTTCTCCTTTCATTTTTTTGTGTGATAAAAGGG
VPS21-3'utr-NotI-R	cgcGCGGCCGCAATATTAACTAATCCATCTCCCGATTTTCG
***YPT52* constructs**	
Not1 Prom Ypt52 fw	gcgcgcggccgcCTGGTAATAACGTCAATACGTACTCTCTTG
HindIII UTR Ypt52 rev	gctcaagcttGCATGGTAAATAGGGTGAACG
HA fw (GS) Ypt52	GGAGTAAACGTATATATTATTAACAGATGTACCCATACGATGTTCCTG
HA rev (GS) Ypt52	CCCAATAAAACTAGTTTAAATTGCAACATAGCGTAATCTGGAACGTCATATGG
Prom Ypt52 rev (GS) HA	CAGGAACATCGTATGGGTACATCTGTTAATAATATATACGTTTACTCC
Ypt52 fw (GS) HA	CCATATGACGTTCCAGATTACGCTATGTTGCAATTTAAACTAGTTTTATTGGG
Ypt52 fw (GS) GFP	GGCATGGATGAACTATACAAAATGTTGCAATTTAAACTAGTTTTATTGGGTG
GFP Ypt52 Fw (GS)	GGAGTAAACGTATATATTATTAACAGatgAGTAAAGGAGAAGAACTTTTCACTGG
GFP Ypt52 rev (GS)	CACCCAATAAAACTAGTTTAAATTGCAACATTTTGTATAGTTCATCCATGCC
Prom Ypt52 GFP (GS) rev	CCAGTGAAAAGTTCTTCTCCTTTACTcatCTGTTAATAATATATACGTTTACTCC

YPT53 **constructs**	
EcoR1 Prom Ypt53 fw	ccccgaattcAGAATTAGGGAGAAGTATTCAAACT
Not1 UTR ypt53 rev	GGGGgcggccgcGCCTTGTGATTCTCGATCTGGCGAT
promYpt53(HA)rev 2	GGAACATCGTATGGGTACATTCTACTTAACTTCGATGCTG
HAtagFW GS HAYpt53 3	CAGCATCGAAGTTAAGTAGAATGTACCCATACGATGTTCC
HAtagREV GS HAYpt53 4	TGTTGGAATAGCTGCTGTATGTTTATCAGCGTAATCTGGAACGTCATATGG
YPT53 FW GS (HA Ypt53) 5	CCATATGACGTTCCAGATTACGCTGATAAACATACAGCAGCTATTCCAACA
utr_ypt53-GFP fw	GCATCGAAGTTAAGTAGAatgAGTAAAGGAGAAGAAC
GFP-YPT53-rev	GGAATAGCTGCTGTATGTTTATCCATTTTGTATAGTTCATCCATGCC
GFP-YPT53-fw	GGCATGGATGAACTATACAAAATGGATAAACATACAGCAGCTATTCC
utr_ypt53-GFP rev	GTTCTTCTCCTTTACTcatTCTACTTAACTTCGATGC
YPT53-orf-EcoRI-F	agctaagaattcATGGATAAACATACAGCAGCTATTCC
YPT53-orf-EcoRI-F	tgcagtaagcttTTAGCAGTTACAACCCCCAG
**chimeric constructs**	
Prom.VPS21-HA53_ F	CCCTTTTATCACACAAAAAAATGTACCCATACGATGTTCCTG
Prom.VPS21-HA53_ R	CAGGAACATCGTATGGGTACATTTTTTTGTGTGATAAAAGGG
Prom.YPT53-HA21_ F	CCATATGACGTTCCAGATTACGCTAACACATCAGTCACTTCCATAAAGTTGG
Prom.YPT53-HA21_ R	CCAACTTTATGGAAGTGACTGATGTGTTAGCGTAATCTGGAACGTCATATGG
YPT53-T(VPS21)_F	GGGAGACTGGGGGTTGTAACTGCTAGAGAGGGGAAAATAAATATATATAAAAAAAAAGTC
YPT53-T(VPS21)_R	GACTTTTTTTTTATATATATTTATTTTCCCCTCTCTAGCAGTTACAACCCCCAGTCTCCC
VPS21-T(YPT53)_F	GCAAACAGTGCTTGCAGTTGTTAGACTGCAAGCAAAACAACAGCAAACG
VPS21-T(YPT53)_R	CGTTTGCTGTTGTTTTGCTTGCAGTCTAACAACTGCAAGCACTGTTTGC
**gene deletions/chromosomal tagging**	
ypt53_KO_F1	AGAATGGATAAACATACAGCAGCTATTCCAACATTAACTATCAAAcggatccccgggttaattaa
ypt53_KO_R1	CAATAGTAGGTTCTTTGCTCTCTTTAAAATCGTCAGATACAAACCgaattcgagctcgtttaaac
ypt52 KO F1	CAGATGTTGCAATTTAAACTAGTTTTATTGGGTGACTCATCCGTGcggatccccgggttaattaa

ypt52 KO R1	GATTGATTGAGATAAAAAAGCAGCACCAATCGTACTTTCACGTAGgaattcgagctcgtttaaac
MUP1 Flag F1	AATATAATCGAACATTACAAAAGTGAACAAGAAAAATCGCTGTACcggatccccgggttaattaa
MUP1 Flag R1	ATAAAGAAAATGCAATTATAAGAATGTTCATACGTGATTATAAGAgaattcgagctcgtttaaac
VPS9_KO_F	TTTCTCTTCATCAAACAACAAAGAACAGGAATCAACAGCCCGGATCCCCGGGTTAATTAA
VPS9_KO_R	TAACTTTTCCATACCTTCCTTGGCATTTCTCATCTTGGAAGAATTCGAGCTCGTTTAAAC
